# Enhanced semantic segmentation in remote sensing images with SAR-optical image fusion (IF) and image translation (IT)

**DOI:** 10.1038/s41598-025-19402-y

**Published:** 2025-10-10

**Authors:** Chongqi Xu, Zhe Geng, Linyi Wu, Daiyin Zhu

**Affiliations:** College of Electronics and Information Engineering, Nanjing University of Aeronautics and Astronautics, Nanjing, 211106 China

**Keywords:** Synthetic aperture radar, Image fusion, Image translation, Land use classification, Mathematics and computing, Optics and photonics

## Abstract

In general, high-fidelity remote sensing requires both synthetic aperture radar (SAR) images, which are available all-day all-weather but could be challenging to interpret, and optical images, which are human-interpretable but are only available in favorable light conditions. Two of the most widely-adopted strategies for combining the complementary information regarding the area of interest revealed in SAR and electro-optical (EO) images are Image Fusion (IF) and Image Translation (IT). IF aims to merge two or more multimodal images into one image, while IT emphasizes on translating the data representations from the images in the source domain to the target domain. Existing methods typically focus on either IF or IT. In this paper, we jointly exploit IF and IT for enhanced semantic segmentation. When the EO image is of high quality, SAR-optical IF is carried out based on NonSubsampled Contourlet Transform and intensity-hue-saturation. When the EO images suffer from heavy noise due to fog/smoke/clouds and SAR images become the last resort, an efficient end-to-end SAR-to-optical IT network based on the diffusion model is adopted. Experimental results show that the proposed DeepLab+IFIT strategy offers an average accuracy (aAcc) of 94.86% and a mean intersection-over-union (mIoU) of 87.11% on the SpaceNet6 dataset, while achieving an aAcc of 95.96% and a mIoU of 80.49% on AIR-MD-SAR-Map dataset, which outperforms several classic semantic segmentation networks.

## Introduction

Although Synthetic Aperture Radar (SAR) can acquire remote sensing images all-day all-weather, these images are usually difficult to interpret by human operators since they defy the Gestalt principle, which is the foundation for human vision. In contrast, although the images acquired by electrical-optical (EO) sensors are more comprehensible to human operators, they are only available when the light condition is favorable. It follows naturally that to acquire high-fidelity remote sensing information regarding the region of interest (ROI), the complementary advantages of the two sensors should be combined. Aiming at this purpose, SAR-optical image fusion (IF) and SAR-optical image translation (IT) are two of the most widely used strategies.

Most IF methods are based on one or two of the following transforms: wavelet transform (WT)^1^, contourlet transform (CT)^2^, and intensity-hue-saturation (IHS) transform^2^. The CT based method performs multi-scale and multi-directional decompositions of the source images, which leads to better shift-invariance property and directional selectivity at the cost of higher computational complexities. The IHS transform divides the optical image into three channels: intensity, hue, and saturation, and the intensity component is substituted with a grayscale stretched SAR image.

The family of conditional generative adversarial network (GAN), which include the CycleGAN^3,4^, the Contrastive Unpaired Translation (CUT) network^5^, and the Unsupervised Generative Attentional Networks for Image-to-image Translation (U-GAT-IT)^6^, has been proved to be a feasible solution to SAR-to-optical IT since 2020. In 2022, several representative GAN-based paired/unpaired SAR-to-optical image translation methods proposed between 2017 and 2021 are compared and evaluated against the SAR2OPT dataset, which consists of SAR images collected with TerraSAR-X at a spatial resolution of 1 m^7^. In 2024, a hybrid conditional GAN is proposed by Han et al., which employs a hybrid generator composed of a CNN branch and a Vision Transformer (ViT) branch to extract and fuse the local and the global features^8^. More recently, the diffusion probabilistic model, which is a parameterized Markov chain trained to produce samples matching the data distribution, has grabbed the attention of many researchers. Specifically, an end-to-end diffusion model (E$^3$Diff) is developed by Qin et al., which exhibited outstanding performance in SAR-to-optical IT mission^9^.

In this paper, we propose the DeepLab+IFIT strategy, which adopts the classic DeepLabv3+ framework as the major network architecture^10^ and jointly exploits IF and IT for enhanced semantic segmentation. When light condition is favorable, SAR-optical IF is carried out based on NonSubsampled Contourlet Transform (NSCT) and IHS. When the quality of the EO image deteriorates due to fog/smoke/clouds and SAR images become the last resort, the E$^3$Diff model is adopted. Unlike the paper authored by Qin et al., which focuses on demonstrating the quality of image translation by evaluating the structural similarity (SSIM) and learned perceptual image patch similarity (LPIPS)^9^, this paper aims to leverage the transformed image for enhanced land-use classification. To validate the effectiveness of the proposed DeepLab+IFIT methodology, we employ two datasets featuring state-of-the-art SAR image resolution (0.5 m): the self-annotated SpaceNet6 (SN6) dataset^11^ and the Airborne Multi-Dimensional Synthetic Aperture Radar Mapping (AIR-MD-SAR-Map) Dataset^12^.

## Methodology

**Fig. 1:**
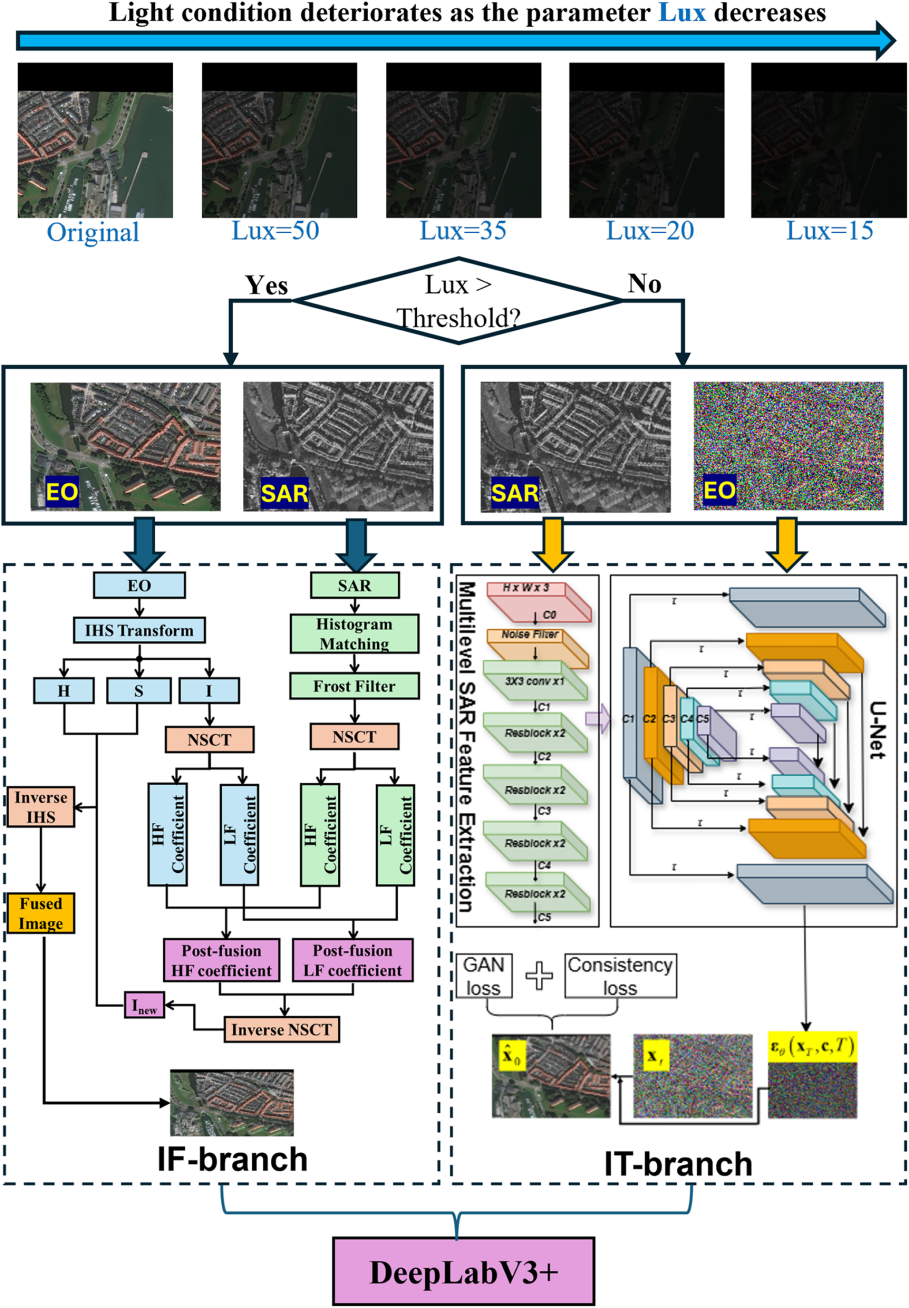
Architecture of the proposed DeepLab+IFIT strategy.

The architecture of the proposed DeepLab+IFIT strategy is illustrated in Fig. 1, which is composed of an IF branch and an IT branch for high-quality and noisy EO images, respectively. Lux is a value measuring illuminance, whose values are extracted from the HSV (Hue, Saturation, Value) images with a mapping function. By jittering the V channel in the HSV images, the lux values could be adjusted. When light condition is favorable, i.e., lux is greater than a predetermined threshold, SAR-optical IF is carried out; otherwise, IT is carried out (the performance of which is not affected by the light condition since only SAR image is used). The IF/IT results are sent to the classic DeepLabv3+ framework, which consists of an encoder and a decoder, for land cover object segmentation^10^.

### IF-branch based on NSCT-IHS transform

It has been demonstrated in our previous work that, for the problem of aircraft and vehicle detection, the contours of the targets, i.e. the high-frequency components, are better reflected in EO images than in SAR images when the light condition is favorable^13^. However, for the problem of land cover segmentation, the SAR images and the EO images are often complimentary in recall and precision depending on the object of interest. For vegetation detection, the EO imagery is more advantageous in recall given that color information is available^14^. But for buildings, road, and water, superior segmentation performance is achievable by jointly exploiting EO and SAR images^10^. From the image frequency spectrum aspect of view, the high-frequency (HF) component mainly captures the region edges and contours, while the low-frequency (LF) component depicts uniform areas featuring gradual changes in intensity^15^. Therefore, the IF-branch is devised based on the principle of adaptive HF-LF feature fusion. With the IHS transform, the optical image is decomposed into the H (“Hue”) channel, the S (“Saturation”) channel, and the I (“Intensity”) channel, i.e.

1 $$\begin{aligned} \left[ \begin{array}{l} {{I}} \\ {{v}_{1}} \\ {{v}_{2}} \\ \end{array} \right]&=\left[ \begin{array}{lll} \frac{1}{3} & \frac{1}{3} & \frac{1}{3} \\ -\frac{\sqrt{2}}{6} & -\frac{\sqrt{2}}{6} & -\frac{2\sqrt{2}}{6} \\ \frac{1}{\sqrt{2}} & -\frac{1}{\sqrt{2}} & 0 \\ \end{array} \right] \left[ \begin{array}{l} R \\ G \\ B \\ \end{array} \right] , \end{aligned}$$

2 $$\begin{aligned} H&= \tan ^{-1}\left( \frac{v_2}{v_1}\right) , \end{aligned}$$

3 $$\begin{aligned} S&= \sqrt{v_1^2+v_2^2}, \end{aligned}$$

where $v_1$ and $v_2$ play the same roles as *x* and *y* axes in the Cartesian coordinate system. Unlike the naive EO-SAR fusion method of substituting the EO data in I channel with the SAR data, we employ the NSCT to further decompose the EO data in I channel and the preprocessed SAR data into HF and LF component. After that, different fusion rules are adopted for these two components to account for their distinctive features. Specifically, LF fusion is based the principle of energy preservation, where the weights are assigned proportionally to the energy of each sub-band in the local region window, i.e.

4 $$\begin{aligned} {\mathbf{LF}}_{new} = w_1 \times {\mathbf{LF}}_{I} + (1-w_1) \times {\mathbf{LF}}_{SAR}, \end{aligned}$$

where $\mathbf{LF}_{I}$ and $\mathbf{LF}_{SAR}$ are the LF components of the intensity component of the EO image and the SAR image obtained using the NSCT transforms, respectively. $w_1$ is defined as the correlation between $\mathbf{LF}_{I}$ and $\mathbf{LF}_{SAR}$, i.e.

5 $$\begin{aligned} w_1 = \frac{\sum {({\mathbf{LF}}_{SAR}-{\overline{\mathbf{LF}}}_{SAR})({\mathbf{LF}}_{I}-{\overline{\mathbf{LF}}_{I})}}}{\sqrt{\sum {({\mathbf{LF}}_{SAR}-\overline{\mathbf{LF}}_{SAR})^2\sum({\mathbf{LF}}_{I}-\overline{{\mathbf{LF}}}_{I})^2}}}, \end{aligned}$$

where $\overline{\mathbf{LF}}_{SAR}$ and $\overline{\mathbf{LF}}_{I}$ represent the mean values of $\mathbf{LF}_{SAR}$ and $\mathbf{LF}_{I}$, respectively. In contrast, HF fusion is based on the principle of edge and fine feature preservation, where the dominant coefficients with greater absolute values are kept so that the fused image exhibits enhanced clarity. The EO-SAR fused intensity component obtained by taking the inverse NSCT, and the reverse transform is given by

6 $$\begin{aligned} \left[ \begin{array}{l} {R} \\ {G} \\ {B} \\ \end{array} \right]&=\left[ \begin{array}{lll} 1 & -\frac{1}{\sqrt{2}} & \frac{1}{\sqrt{2}} \\ 1 & -\frac{1}{\sqrt{2}} & -\frac{1}{\sqrt{2}} \\ 1 & {\sqrt{2}} & 0 \\ \end{array} \right] \left[ \begin{array}{l} {{I}_{new}} \\ {{v}_{1}} \\ {{v}_{2}} \\ \end{array} \right] \end{aligned}$$

### IT-branch based on the diffusion model

The main architecture of the IT branch is based on the end-to-end diffusion model (E$^3$Diff) developed by Qin et al. for SAR-to-optical IT, where the multilevel SAR prior features are used to control U-Net for denoising, and the iterative denoising process of diffusion is transformed into one-step deterministic mapping by treating the generative process as the reverse of a particular Markovian diffusion process^9^. Diffusion models are latent variable models with density function given by $p_\theta (\mathbf{x}_0) = \int p_\theta (\mathbf{x}_{0:T}) \,d\mathbf{x}_{1:T}$, where $\mathbf{x}_1, \dotsc , \mathbf{x}_T$ are latents of the data $\mathbf{x}_0$. The joint distribution is given by

7 $$\begin{aligned} p_\theta (\mathbf{x}_{0:T}) = p(\mathbf{x}_T)\prod _{t=1}^T p_\theta (\mathbf{x}_{t-1}|\mathbf{x}_t). \end{aligned}$$

For simplicity, the reverse process is defined as Gaussian distribution with mean ${\varvec{\mu }}_\theta (\mathbf{x}_t, t)$ and variance ${\varvec{\Sigma }}_\theta (\mathbf{x}_t, t) = \sigma _t^2 \mathbf{I}$, i.e.:

8 $$\begin{aligned} p_\theta (\mathbf{x}_{t-1}|\mathbf{x}_t) = \mathcal {N}(\mathbf{x}_{t-1}; {\varvec{\mu }}_\theta (\mathbf{x}_t, t),\sigma _t^2 \mathbf{I} ), \end{aligned}$$

**Fig. 2:**
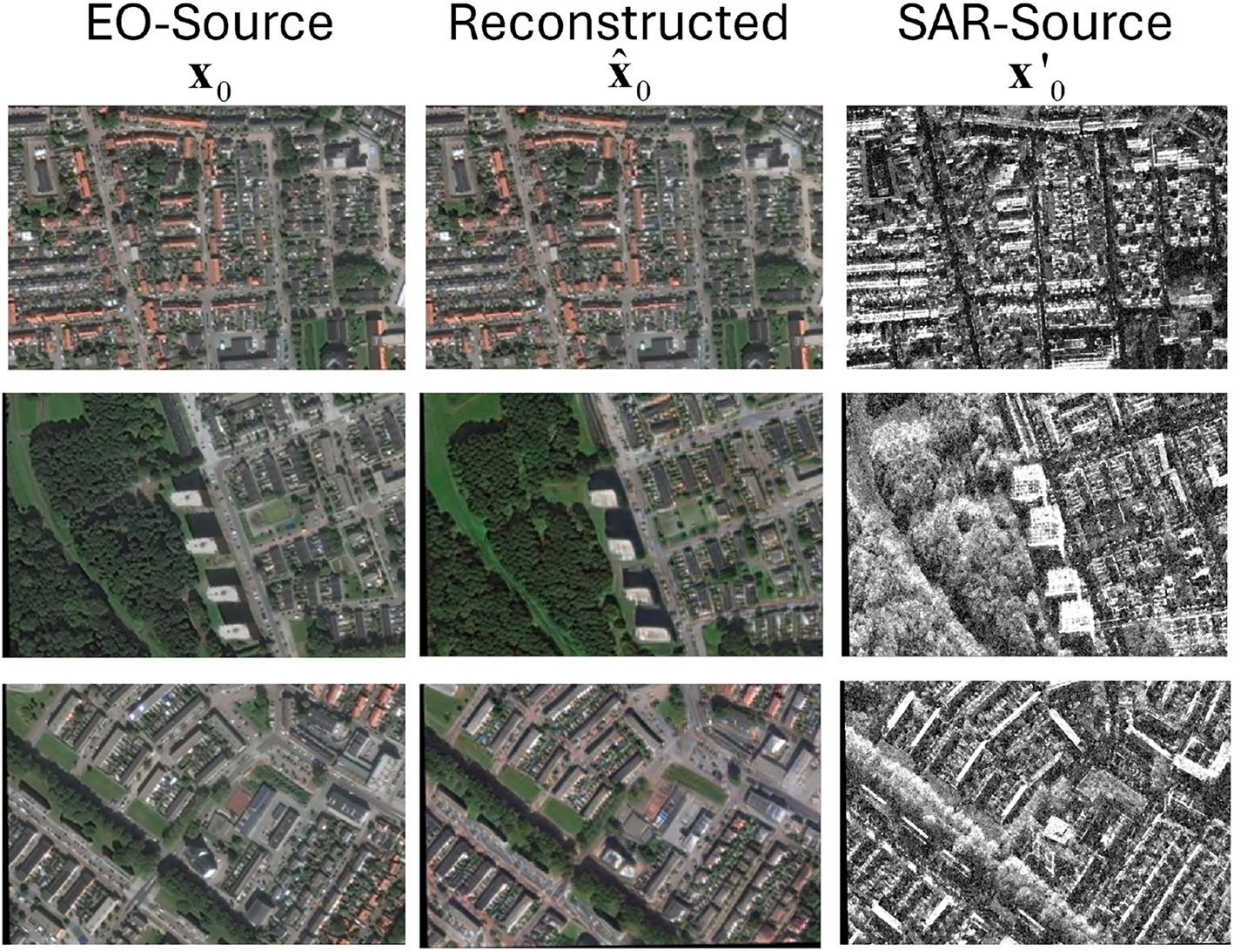
The EO source image $\mathbf{x}_0$ and the EO image $\hat{\mathbf{x}}_0$ reconstructed by the diffusion model based on the multilevel features extracted from the SAR source image $\mathbf{x}'_0$.

For the forward process, the approximate posterior

9 $$\begin{aligned} q(\mathbf{x}_{1:T} | \mathbf{x}_0) = \prod _{t=1}^T q(\mathbf{x}_t | \mathbf{x}_{t-1} ). \end{aligned}$$

is modeled by a Markov chain which adds Gaussian noise to the data gradually according to variance schedule $\beta _1, \dotsc , \beta _T$. Using the notation $\alpha _t = 1-\beta _t$ and $\bar{\alpha }_t = \prod _{s=1}^t \alpha _s$, we have^16^

10 $$\begin{aligned} q(\mathbf{x}_t|\mathbf{x}_0) = \mathcal {N}(\mathbf{x}_t; \sqrt{\bar{\alpha }_t}\mathbf{x}_0, (1-\bar{\alpha }_t){\mathbf{I}}), \end{aligned}$$

which is equivalent to $\mathbf{x}_t(\mathbf{x}_0, {\varvec{\varepsilon }}) = \sqrt{\bar{\alpha }_t}\mathbf{x}_0 + \sqrt{1-\bar{\alpha }_t}{\varvec{\varepsilon }}$ for ${\varvec{\varepsilon }} \sim \mathcal {N}({\mathbf{0}}, {\mathbf{I}})$. The EO source image $\mathbf{x}_0$ and the synthesized EO image $\hat{\mathbf{x}}_0$ generated by the diffusion model based on the SAR source image $\mathbf{x}'_0$ illustrated in Fig. 2.

$\hat{\mathbf{x}}_0$ is obtained with

11 $$\begin{aligned} {\hat{\varvec{x}}_{0}} = \frac{ \sqrt{\bar{\alpha }_{0}} {{\varvec{x}}_{T}} }{\sqrt{\bar{\alpha }_{T}}} + \left( {{ {\sqrt{1-\bar{\alpha }_{0}}} - \frac{\sqrt{\left( {{1-\bar{\alpha }_{T}}}\right) \bar{\alpha }_{0}} }{\sqrt{\bar{\alpha }_{T}}} }}\right) {\varvec{\varepsilon }}_{\theta }\left( {{{\varvec{x}}_{T}, {\varvec{c}}, T}}\right) . \end{aligned}$$

where ${\varvec{c}}$ is the multilevel features of the SAR spatial prior extracted from the SAR source image $\mathbf{x}'_0$, and ${\varvec{\varepsilon }}_{\theta }\left( {{{\varvec{x}}_{T}, {\varvec{c}}, T}}\right)$ is a function approximation for ${\varvec{\varepsilon }}$, which is to be learned from the training data. The refinement loss is defined as

12 $$\begin{aligned} {{\mathcal {L}}} = {{\mathcal {L}}}_{\textrm{ Simple}}+ {{\mathcal {L}}}_{C} +\lambda _{1}{{\mathcal {L}}}_{\textrm{ GAN}}, \end{aligned}$$

where ${{\mathcal {L}}}_{\textrm{ Simple}}$, ${{\mathcal {L}}}_{C}$, and ${{\mathcal {L}}}_{\textrm{ GAN}}$ represent the approximation loss between ${\varvec{\varepsilon }}_{\theta }\left( {{{\varvec{x}}_{T}, {\varvec{c}}, T}}\right)$ and ${\varvec{\varepsilon }}$, the consistency loss, and the GAN loss, respectively; $\lambda _1$ is a parameter controlling the weight of ${{\mathcal {L}}}_{\textrm{ GAN}}$ and is set as 0.5 empirically^9^. ${{\mathcal {L}}}_{\textrm{ Simple}}$ in (12) is given by^9^

13 $$\begin{aligned} {{\mathcal {L}}}_{\textrm{ Simple}} = \mathbb {E}_{{\varvec{x}}_{0},t, {\varvec{\epsilon }}}\left( {{\Vert {\varvec{\epsilon }}_{\theta }\left( {{ {\varvec{x}}_{0},{\varvec{c}}, {t}}}\right) -{\varvec{\epsilon }} \Vert ^{2}}}\right) , \end{aligned}$$

which is to be minimized to ensure the sample quality. The consistency loss ${{\mathcal {L}}}_{C}$ in (12) consists of two parts to account for pixel-level and global-level consistency and is given by^9^

14 $$\begin{aligned} {{\mathcal {L}}}_{C} = \mathbb {E}_{{\varvec{x}}_{0}, \hat{\varvec{x}}_{0}} \left( {{ \Vert {{\varvec{x}}_{0}-\hat{\varvec{x}}_{0}} \Vert ^{2} }}\right) + \lambda _{2} \mathbb {E}_{{\varvec{x}}_{0}, \hat{\varvec{x}}_{0}} \left( {{ \Vert F\left( {{ {\varvec{x}}_{0} }}\right) - F\left( {{ \hat{\varvec{x}}_{0} }}\right) \Vert ^{2} }}\right) , \end{aligned}$$

where *F* is the network for image feature extraction; $\lambda _2$ is a parameter to control the weight of pixel-level and global-level consistency and is set as 5 by default^9^. The GAN loss ${{\mathcal {L}}}_{\textrm{ GAN}}$ in (12) is given by

15 $$\begin{aligned} \mathcal {L}_{\text {GAN}} = \mathbb {E}_{y \sim p_{\text {data}}(y)}[\log D_Y(y)] + \mathbb {E}_{x \sim p_{\text {data}}(x)}[\log (1-D_Y(G(x))], \end{aligned}$$

where $\{x_i\}_{i=1}^N$, $x_i \in X$ and $\{y_j\}_{j=1}^M$, $y_j \in Y$ are the training samples from the source domain *X* and the target domain *Y*, respectively, with *M* and *N* represent the number of samples; $G: X\rightarrow Y$ is the mapping function; $x\sim p_{data}(x)$ and $y\sim p_{data}(y)$ represent the data distribution; $D_Y$ is the discriminator. During the one-step reverse sampling process described in (11), ${{\mathcal {L}}}_{\textrm{ Simple}}$, ${{\mathcal {L}}}_{C}$ , ${{\mathcal {L}}}_{\textrm{ GAN}}$ are jointly optimized to minimize ${{\mathcal {L}}}$ in (12).

The EO-SAR fusion result generated by the IF-branch based on the NSCT-IHS transform and the reconstructed EO image generated by the E$^3$Diff model in the IT-branch conditioned on the multilevel SAR features are then used as inputs for DeepLabv3+ to realize land-use segmentation.

## Experiments

**Fig. 3:**
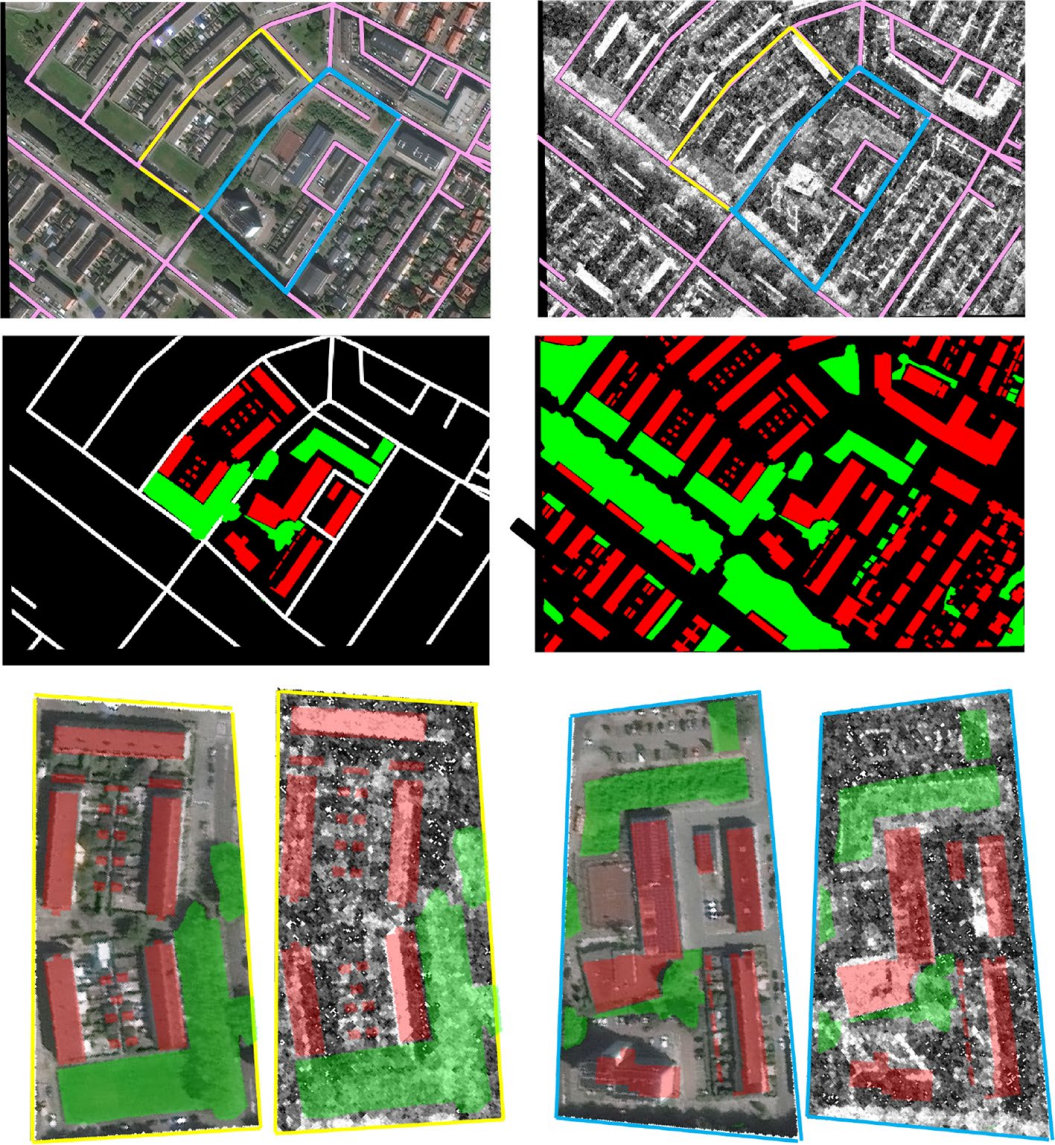
An example of road-guided neighborhood recognition supported by high-resolution EO-SAR image pairs.

To validate the effectiveness of the proposed methodology, we employ two datasets (1) the SpaceNet6 (SN6) dataset^11^ with semantic labels for buildings and vegetation added manually; and (2) the AIR-MD-SAR-Map Dataset, which consists of 9 categories of land cover objects^12^. The SAR and EO images from these datasets feature a resolution of 0.5 m, 10 times finer than the first and largest joint optical and SAR land use classification dataset, the WHU-OPT-SAR dataset presented in 2022^10^. Since imagery interpretability is heavily influenced by image resolution, we believe the results presented in this paper is of great significance to the research in joint context-target analysis such road-guided neighborhood recognition (see Fig. 3), and the detection of vehicles in a parking lot or on a typical road, which requires a resolution of at least 0.5 m to accomplish.

### Experimental results on the SN6 dataset

**Fig. 4:**
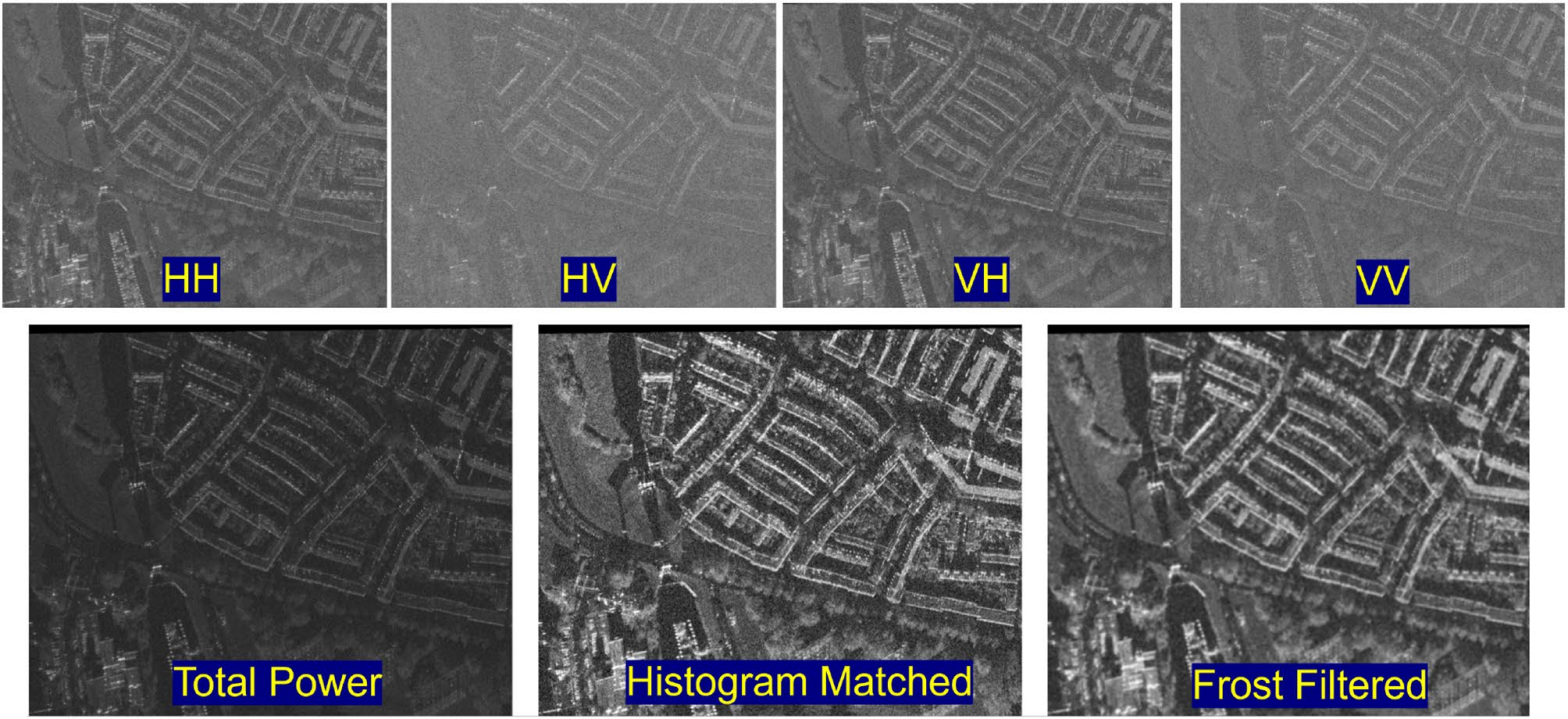
The original quad-polarized SAR imagery and the image preprocessing result.

The SN6 dataset consists of quad-polarized (HH, HV, VH, VV) X-band SAR imagery of 0.5 m resolution and optical imagery of 0.5 m resolution over the port of Rotterdam, the Netherlands. It covers $120\;{\text{km}}^{2}$ and is labeled with 48000 building footprints. To fully investigate the impact of the characteristic difference in land-cover objects, we labeled the vegetation area manually. In image preprocessing, the total power received by the four channels of the polarimetric radar system is calculated. The total-power image, the histogram-matching result, and the Frost-filtered image are shown in Fig. 4.

For the IT-branch based on the diffusion model, the training-test split ratio for the SN6 dataset is set as 8:2, which results in 2701 and 680 EO-SAR image pairs for training and test, respectively. The 900$\times$900 input images are cropped into 256$\times$256 chips. *T* in (7) is set as 1000, while the forward process variances are set as constants increasing linearly from $\beta _1 = 10^{-6}$ to $\beta _T = 0.01$. The experiment is carried out with the following settings: batch size = 4, learning rate = $5\times 10^{-5}$, maximum epoch = $10^5$, and Adam optimizer. In the image segmentation stage, the experimental settings are: batch size = 4, Stochastic Gradient Descent (SGD) optimizer with momentum of 0.9 and weight decay of $5\times 10^{-4}$, PolyLRScheduler in the timm library with an initial learning rate of 0.01 (learning rate of each parameter group decays according to a polynomial function in the total number of steps set by the user), data augmentation with the probability of random image flipping set as 0.5, and maximum epoch = $8\times 10^4$.

**Table 1:** Segmentation performance of the proposed DeepLab-IFIT strategy on the SN6 dataset (%).

Data modality	Model	Segmentation performance				
		Background	Buildings	Vegetation	aAcc	mIoU
EO-Lux15	DeepLabv3+r50	86.59	65.08	75.61	89.92	75.76
	DeepLabv3+r101	86.97	63.17	76.79	90.24	75.64
EO-Lux20	DeepLabv3+r50	89.98	76.46	81.38	92.78	82.60
	DeepLabv3+r101	90.34	76.80	81.95	93.05	83.03
EO-Lux35	DeepLabv3+r50	91.68	81.41	84.05	94.09	85.71
	DeepLabv3+r101	91.79	81.94	84.41	94.29	86.11
EO-Lux50	DeepLabv3+r50	91.91	82.23	84.31	94.26	86.15
	DeepLabv3+r101	92.19	82.76	84.71	94.46	86.55
EO-Original	DeepLabv3+r50	92.02	82.59	84.45	94.34	86.35
	DeepLabv3+r101	92.33	83.26	84.92	94.56	86.83
SAR	DeepLabv3+r50	89.12	69.53	81.40	92.04	80.02
	DeepLabv3+r101	89.82	70.98	82.77	92.59	81.19
SAR-SPAN	DeepLabv3+r50	89.63	70.77	82.41	92.46	80.94
	DeepLabv3+r101	90.27	72.29	83.71	92.96	82.09
EO-SAR (IT)	DeepLabv3+r50	91.27	72.12	86.79	93.72	83.40
	DeepLabv3+r101	91.67	73.00	87.51	94.02	84.06
EO-SAR (IF)	DeepLabv3+r50	92.36	81.02	85.99	94.58	86.46
	DeepLabv3+r101	92.73	81.82	86.78	94.86	87.11

**Fig. 5:**
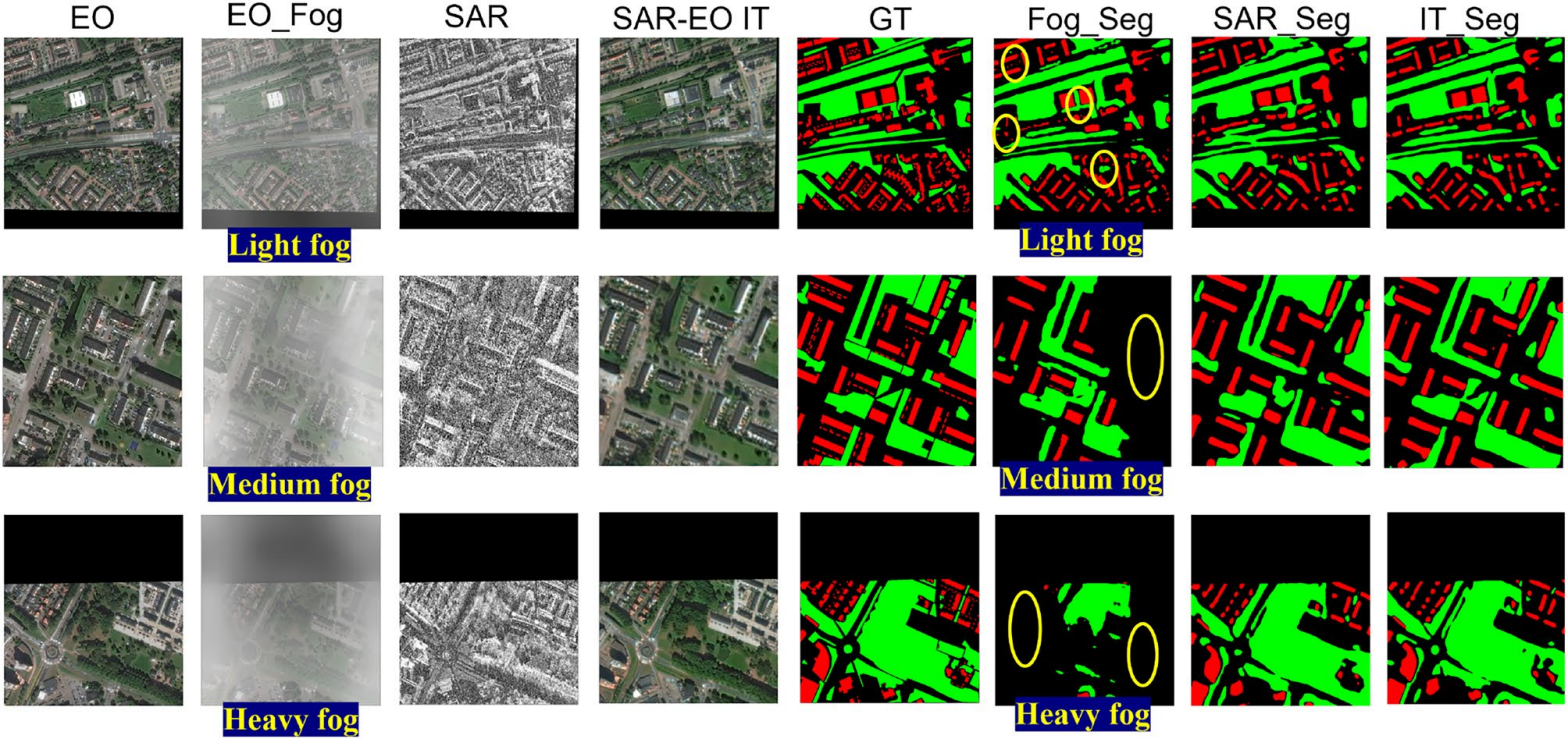
Segmentation results for some representative images in the SN6 dataset. Green, red, and black represent vegetation, building, and background, respectively.

The segmentation performance of the proposed DeepLab-IFIT strategy on the preprocessed images from the SN6 dataset under different light conditions is summarized in Table 1, where the mean intersection over union (mIOU) and average accuracy (aAcc) are evaluated. Some exemplar images correspond to $lux = 15, 20, 35, 50$ could be found in Fig. 1. The number of image samples used for training and test are 2240 and 560, respectively, and the image size is $900 \times 900$. To further demonstrate the robustness of the proposed DeepLab+IFIT strategy across varying level of visibility, the segmentation results for some representative images in the SN6 dataset under the condition of light fog, medium fog, and heavy fog are presented in Fig. 5. It could be seen that (1) moderate-thick fog could have devastating impact on segmentation results based solely on optical images; and (2) the segmentation results generated by the proposed strategy (i.e. ITSeg) are closer to the ground truth in the circled areas than those generated based solely on SAR images.

### Experimental results on the AIR-MD-SAR-Map dataset

**Fig. 6:**
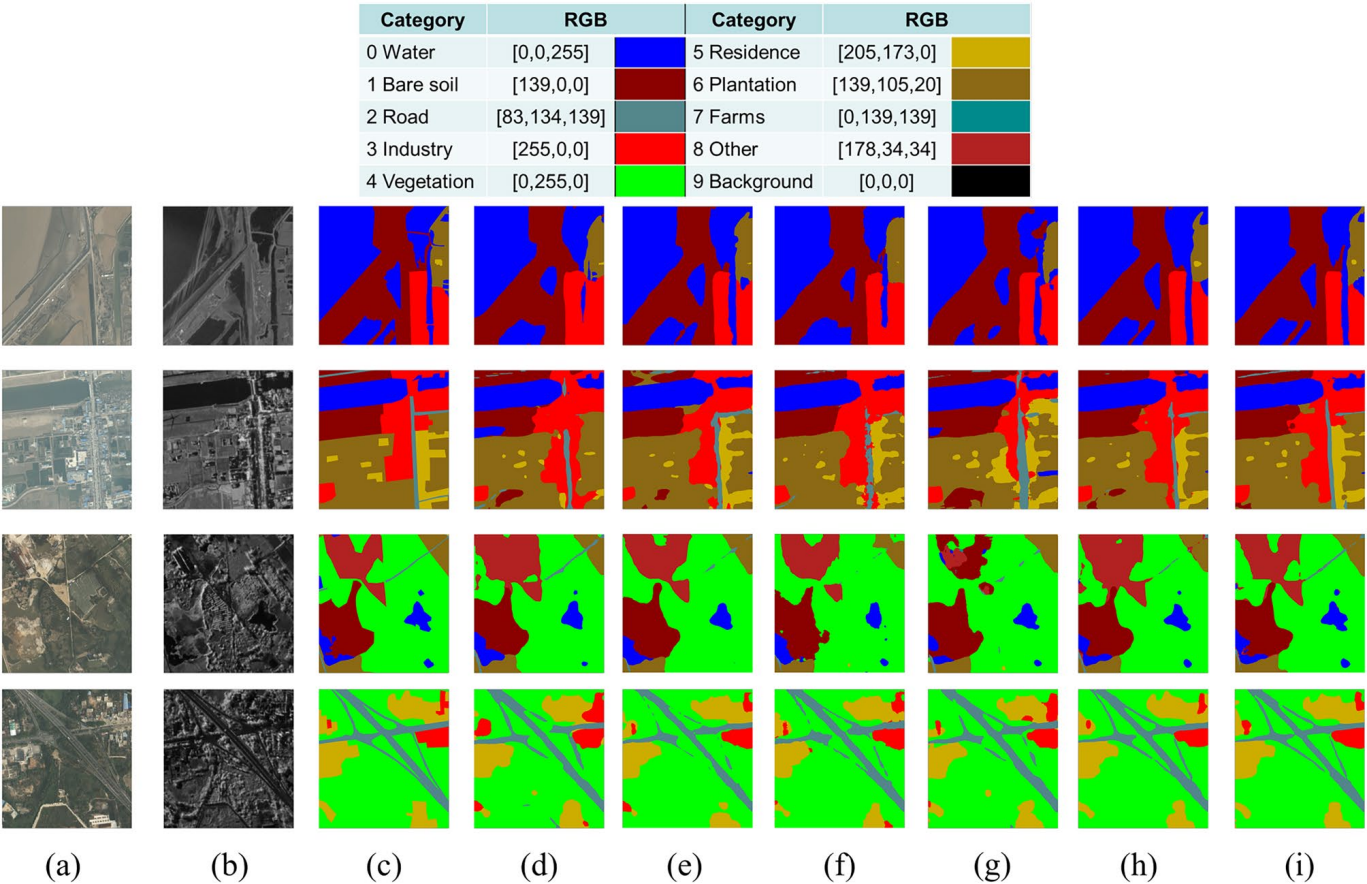
Performance comparison between the segmentation results provided by the proposed DeepLab-IFIT strategy and several representative semantic segmentation networks on some image samples in the AIR-MD-SAR-Map dataset. (**a**) EO images. (**b**) SAR images. (**c**) Ground truth. (**d**) PSPNet classification results. (**e**) UPerNet classification results. (**f**) SegFormer classification results. (**g**) DeepLab3+r18 classification results. (**h**) deeplabv3+r50 classification results. (**i**) EO-SAR (IF) classification results.

The AIR-MD-SAR-Map dataset consists of quad-polarized airborne SAR images in bands of C, Ka, L, P, and S and high-resolution optical images in Hainan, China and Jiangsu, China, with the spatial resolution ranging from 0.2 to 1 m depending on the band. The Ka-band images with 0.5 m resolution are used as the experimental data for this section. The experiment on the AIR-MD-SAR-Map dataset is carried out with the same parameter settings used for the experiment on the SN6 dataset. The segmentation performance and the computational complexity (evaluated by floating point operations per second, or FLOPs) of the proposed DeepLab-IFIT strategy on the AIR-MD-SAR-Map dataset based on the DeepLabv3+r101 structure is compared with Pyramid Scene Parsing Network (PSPNet)^17^, Unified Perceptual Parsing UPerNet^18^, SegFormer^19^, DeepLabv3+r18, and DeepLabv3+r50 in Table 2, where Cls0-Cls9 represent water, bare soil, road, industry, vegetation, residence, plantation, farms, other, and background, respectively. The number of training and test samples are 1356 and 339, respectively. As is shown in Table 2, the proposed DeepLab-IFIT strategy exhibits superior performance in the aspect of mIOU and aAcc at a cost of slightly higher computational complexity. The segmentation results of these networks for some representative images in the AIR-MD-SAR-Map dataset are presented in Fig. 6. It could be seen from Fig. 6 that the proposed DeepLab-IFIT strategy offers the best segmentation performance.

**Table 2:** Segmentation performance of the proposed DeepLab-IFIT strategy on the AIR-MD-SAR-Map dataset (%).

Model	Segmentation performance												Complexity	
	cls0	cls1	cls2	cls3	cls4	cls5	cls6	cls7	cls8	cls9	aAcc	mIou	Flops (G)	Param (M)
PSPNet	87.25	77.15	53.73	80.44	84.91	72.83	87.68	71.71	13.67	99.89	93.24	72.93	54.356	12.640
UPerNet	89.95	82.80	58.74	85.62	87.36	76.84	90.47	76.24	15.83	99.93	94.61	76.38	237.058	64.046
SegFormer	89.98	84.84	58.06	86.23	87.86	77.27	91.29	79.19	16.08	99.93	94.88	77.07	6.157	2.471
DeepLabv3+r18	86.62	75.24	53.85	79.57	83.80	71.94	86.68	70.88	6.21	99.90	92.79	71.47	54.356	12.319
DeepLabv3+r50	91.31	84.56	66.23	89.50	89.73	81.57	92.02	84.02	18.11	99.92	95.56	79.70	176.918	41.221
EO-SAR (IF)	93.37	86.62	69.81	90.33	90.33	82.33	92.80	82.61	16.97	99.87	95.96	80.49	254.923	60.213

## Conclusion

A dual-branch land-use segmentation framework, DeepLab+IFIT, is proposed, which consists of an IF branch for EO-SAR image fusion given that the EO images are of high quality, and an IT branch for EO image reconstruction conditioned on the multilevel features extracted from the SAR images in case of heavy noise. The performance of the DeepLab+IFIT framework is tested against two datasets, the SN6 dataset consisting of X-band satellite-borne SAR images and the AIR-MD-SAR-Map dataset consisting of Ka-band airborne SAR images. Experimental results show that the proposed DeepLab+IFIT strategy offers an average accuracy (aAcc) of 94.86% and a mean intersection-over-union (mIoU) of 87.11% on the SpaceNet6 dataset, while achieving an aAcc of 95.96% and a mIoU of 80.49% on AIR-MD-SAR-Map dataset, which outperforms several classic semantic segmentation networks. By demonstrating the effectiveness of the proposed DeepLab+IFIT framework against state-of-the-art SAR images featuring 0.5 m-resolution, which is 10 times finer than the first joint optical-SAR land-use dataset presented in 2022, this paper lays the foundation for futuristic joint context-target analysis research such as road-guided neighborhood recognition and parking-lot/road-guided vehicle detection.

## Data Availability

The research data used for the experiments in this paper can be found in https://medium.com/the-downlinq/spacenet-6-expanded-dataset-release-e1a7ddaf030 and https://huggingface.co/datasets/damonzheng/AIR-MDSAR-Map/tree/main.
